# Association of inflammatory markers with functional outcome after endovascular thrombectomy in anterior circulation stroke: a retrospective study

**DOI:** 10.3389/fneur.2026.1808319

**Published:** 2026-09-11

**Authors:** Yun Mo, Xue Guan, Yuxuan He, Lijuan Wu, Meng Zuo, Zhenhua Zhou

**Affiliations:** 1 Department of Neurology, Southwest Hospital, Third Military Medical University (Army Medical University), Chongqing, China; 2 Department of Neurology, The First People's Hospital of Wanzhou District, Chongqing, China

**Keywords:** acute ischemic stroke, endovascular thrombectomy, inflammatory indicators, large vessel occlusion, symptomatic intracranial hemorrhage

## Abstract

**Background:**

Endovascular thrombectomy (EVT) is an effective treatment for acute ischemic stroke (AIS) caused by large vessel occlusion in the anterior circulation. However, functional outcomes vary. Systemic inflammatory response may affect prognosis. This study aims to determine the prognostic association of inflammatory indicators for favorable outcomes in patients undergoing EVT.

**Method:**

In this retrospective study, 378 patients with anterior circulation large vessel occlusion stroke (LVOS) who underwent EVT were included. Patients were stratified into favorable outcome group (mRS ≤ 2) and unfavorable outcome group (mRS > 2). Clinical baseline data and laboratory blood tests were completed on admission. Imaging data were collected between 2 and 5 days after admission. Univariate and multivariate analyses were performed to evaluate the factors associated with favorable outcome.

**Result:**

Compared to the favorable outcome group, the unfavorable outcome group had a higher incidence of symptomatic intracranial hemorrhage (sICH) (22%) and stroke-associated pneumonia (SAP) (59%), lower successful recanalization (mTICI 2b-3: 83.7%), and poorer collateral circulation status (58.8%). Furthermore, the unfavorable outcome group exhibited significantly higher preoperative white blood cell count, neutrophil count, monocyte count, and neutrophil-to-lymphocyte ratio (NLR) (all *p* < 0.05), while the lymphocyte-to-monocyte ratio (LMR) and platelet distribution width (PDW) were significantly lower (all *p* < 0.05). The AUC for LMR was 0.578 (95% CI 0.517–0.639), with an optimal cut-off value of 2.69 (sensitivity 64.7%, specificity 48.8%). The AUC for PDW was 0.572 (95% CI 0.511–0.636), with an optimal cut-off value of 15.2% (sensitivity 78.9%, specificity 43.1%).

**Conclusion:**

Inflammatory indicators, particularly LMR and PDW, were associated with functional outcome after EVT in patients with anterior circulation large vessel occlusion and may provide supplementary information for risk stratification.

## 1 Introduction

Acute ischemic stroke (AIS) is one of the leading global causes of disability and mortality. Endovascular thrombectomy (EVT) has been shown to significantly improve recanalization rates and clinical outcomes (1–3). However, despite advances in recanalization techniques, about half of patients still fail to achieve favorable functional outcomes. This suggests that other critical pathophysiological processes play a vital role in post-EVT neurological recovery (4).

In recent years, secondary injury mechanisms following EVT have garnered increasing attention, with immune-inflammatory responses recognized as a key contributor. Inflammatory cells, such as neutrophils and monocytes/macrophages, are rapidly activated and infiltrate the ischemic brain tissue, releasing large quantities of reactive oxygen species, proteases, and pro-inflammatory cytokines that exacerbate blood–brain barrier disruption and cerebral edema (5). Concurrently, a systemic immunosuppressive state may predispose patients to complications such as stroke-associated pneumonia (SAP), further worsening prognosis (6).

Consequently, there is an urgent clinical need to identify accessible biomarkers capable of accurately reflecting the systemic inflammatory status and immune balance, which would facilitate individualized risk stratification and outcome prediction for patients undergoing EVT.

The present study aims to comprehensively investigate the associations between preoperative serum inflammatory indices, and post-EVT functional outcomes, in patients with anterior circulation large vessel occlusion (AC-LVO).

## 2 Method

### 2.1 Ethical approval

This study collected clinical and imaging data from hospitalized patients with AC-LVO who underwent EVT between January 2017 and October 2021. The study protocol was approved by the Ethics Review Boards of Southwest Hospital and the Third People’s Hospital of Zigong [(B)KY2021146 and (2021-01-01)]. Written informed consent for EVT was obtained from all participating patients. In this retrospective analysis, all clinical and imaging data were anonymized and de-identified prior to analysis to ensure patient privacy. Given that the data analysis was non-interventional, the ethics committee waived the requirement for additional informed consent for this retrospective study.

### 2.2 Inclusion and exclusion criteria

Inclusion criteria were as follows: (1) age > 18 years; (2) definitive diagnosis of AIS; (3) confirmation of AC-LVO by computed tomography angiography (CTA), magnetic resonance angiography (MRA), or intraoperative digital subtraction angiography (DSA) AC-LVO in this study was defined as occlusion of the intracranial internal carotid artery (ICA) and/or the M1/M2 segment of the middle cerebral artery; isolated anterior cerebral artery occlusion and bilateral large-vessel occlusion were excluded; (4) treatment initiation within 6 h of symptom onset, or for patients presenting between 6 to 24 h, presence of clinical deficit-infarct volume mismatch based on magnetic resonance imaging.

Exclusion criteria were as follows: (1) posterior circulation infarction; (2) life expectancy of less than 3 months; (3) haematologic disease; (4) incomplete clinical or imaging data.

**Figure 1:**
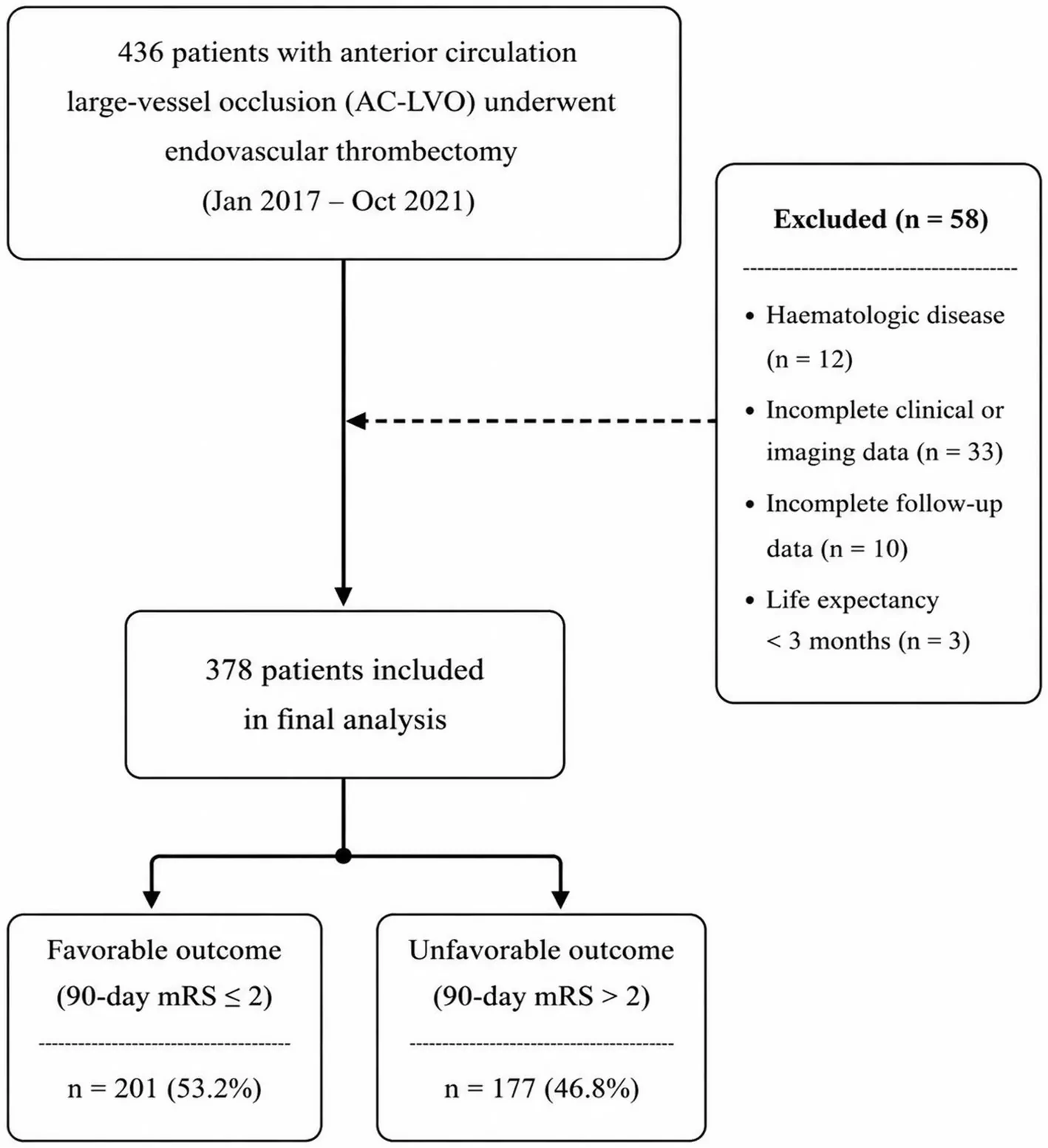
Patient flow diagram. AC-LVO, anterior circulation large-vessel occlusion; mRS, modified Rankin Scale.

During the study period (January 2017–October 2021), 436 patients with anterior circulation large-vessel occlusion underwent endovascular thrombectomy at the participating centers. Of these, 12 were excluded for pre-existing haematologic disease, 33 for incomplete clinical or imaging data, 10 for incomplete follow-up data, and 3 for a life expectancy of less than 3 months. This yielded a final analytic cohort of 378 patients. The patient selection process is summarized in Figure 1. No formal *a priori* sample size calculation was performed, as this was a retrospective analysis of all eligible patients treated during the study period.

### 2.3 Diagnostic criteria

Symptomatic intracranial hemorrhage (sICH) was defined as any intracranial hemorrhage (including parenchymal hematoma, subarachnoid, or intraventricular hemorrhage) associated with an increase of ≥4 points in the National Institutes of Health Stroke Scale (NIHSS) score (7).

SAP was defined according to previously established research consensus, as a lower respiratory tract infection occurring within the first 7 days after stroke onset (6, 8).

### 2.4 Data collection

We collected patients’ baseline characteristics, including demographic features (sex, age), vascular risk factors (hypertension, dyslipidemia, diabetes, atrial fibrillation, prior stroke, smoking), NIHSS score on admission, stroke etiology classified according to the Trial of Org 10172 in Acute Stroke Treatment (TOAST) criteria, stent implantation, and intravenous thrombolysis. Functional outcome was assessed using the mRS at 90 days, with an mRS score ≤2 defined as a favorable outcome and an mRS score ≥3 defined as an unfavorable outcome. All patients are required to undergo cranial CT or MRI examinations upon admission and after EVT, and all imaging data are analyzed by two experienced radiology experts. Imaging was independently reviewed for the purposes of this study by two radiologists, and inter-rater agreement was assessed using Cohen’s κ coefficient: κ = 0.819 (95% CI 0.723–0.900, *p* < 0.001) for symptomatic intracerebral hemorrhage, κ = 0.748 (95% CI 0.626–0.852, *p* < 0.001) for mTICI 2b/3 reperfusion status, and κ = 0.699 (95% CI 0.624–0.771, *p* < 0.001) for ASITN/SIR <2 collateral grade, indicating substantial to excellent agreement. Any discrepancies between the two readers were resolved by adjudication from a third, more senior radiologist. The vascular recanalization status is evaluated using the modified Thrombolysis in Cerebral Infarction (mTICI) grading system, with 0-2a considered ineffective recanalization and 2b-3 defined as effective recanalization (9). The assessment of collateral circulation is based on the scoring criteria of the American Society of Interventional and Therapeutic Neuroradiology/Society of Interventional Radiology (ASITN/SIR), with ASITN/SIR <2 indicating poor collateral circulation. Peripheral venous blood samples were collected from all patients within 24 h of admission and prior to the initiation of endovascular thrombectomy. Venous blood samples are collected on admission by a professional laboratory physician at emergency room, who provides results including routine blood tests, low-density lipoprotein, blood glucose, homocysteine, and creatinine, and calculates the systemic immune-inflammation index (SII) based on neutrophil count, lymphocyte count, neutrophil-to-lymphocyte ratio (NLR), and platelet count (10).

### 2.5 Statistical analysis

All continuous data are expressed as mean ± standard deviation; data not following a normal distribution are expressed as median; *t*-test or *Z*-test is used for continuous data; non-continuous data are expressed as percentages and analyzed using the chi-square test; logistic regression is used to calculate the association between lymphocyte-to-monocyte ratio (LMR), platelet distribution width (PDW) and patient functional prognosis.

Continuous data are presented as mean ± standard deviation or median, and compared using *t*-test or *Z*-test. Categorical variables are expressed as percentages and compared using the chi-square test. The impact of LMR and PDW on the prognosis of patients with acute cerebral infarction with anterior circulation occlusion was evaluated by logistic regression analysis. All *p*-values are two-sided, and *p* < 0.05 was considered statistically significant. All analyses were performed using SPSS version 22.0 (SPSS Inc., Chicago, IL, USA) and Python (version 3.8; Python Software Foundation, Wilmington, DE, USA).

In the logistic regression analysis, favorable functional outcome (mRS ≤ 2 vs. mRS > 2 at 90 days) was defined as the dependent variable. Variables that differed significantly between the favorable and unfavorable outcome groups on univariate analysis were considered candidate independent variables; because white blood cell count, neutrophil count, monocyte count and NLR are mathematically or biologically collinear with LMR, only LMR and PDW were entered into the final multivariable model to avoid multicollinearity. Univariate logistic regression was first performed for each candidate variable, followed by multivariable logistic regression including LMR and PDW simultaneously. To further characterize the discriminative performance of LMR and PDW, receiver operating characteristic (ROC) curve analysis was performed, and the area under the curve (AUC), optimal cut-off value (maximum Youden index), sensitivity, and specificity were calculated. Internal validation of the multivariable model was performed using bootstrap resampling (2,000 resamples with replacement) to evaluate the stability of the odds ratio estimates. As a sensitivity analysis, given the marked between-group imbalance in recanalization and collateral circulation status, the association between LMR/PDW and functional outcome was re-examined after additional adjustment for mTICI recanalization status, ASITN/SIR collateral grade, age, and baseline NIHSS score. As an additional robustness check, a stratified analysis (by recanalization status and collateral grade) was also performed. Inter-rater agreement between the two independent radiological readers was quantified using Cohen’s κ coefficient.

## 3 Result

### 3.1 Baseline data and univariate analysis

A total of 378 patients were enrolled in this study. Baseline characteristics stratified by mRS scores are presented in Table 1. There were 186 males (49.2%) and 192 females (50.8%), with 201 patients (53.2%) classified as having favorable outcomes and 177 patients (46.8%) as having unfavorable outcomes. Among them, 51 patients (13.5%) experienced sICH and 166 patients (43.9%) developed SAP.

**Table 1:** Baseline characteristics.

Variable	Favorable outcome (*n* = 201)	Unfavorable outcome (*n* = 177)	*t*, *Z or X^2^*	*p* value
Demographic data				
Age (year, x̄ ± s)	69.52 ± 12.04	71.23 ± 11.95	1.382^a^	0.168
Male (*n*, %)	96 (47.8)	90 (50.8)	0.359	0.549
OTR (min, M, IQR)	360.00 (275.50–527.50)	400.00 (300.00–510.00)	−1.263	0.207
OTP (min, M, IQR)	255.00 (192.50–448.00)	277.50 (212.50–360.00)	−0.051	0.960
Vascular risk factors, (*n,* %)				
Hypertension	106 (52.7)	93 (52.5)	0.001	0.970
Hyperlipidaemia	13 (6.5)	17 (9.6)	1.268	0.265
Diabetes mellitus	38 (18.9)	38 (21.5)	1.76	0.535
Atrial fibrillation	84 (48.8)	84 (49.7)	2.385	0.123
Previous stroke	22 (10.9)	16 (9.0)	0.378	0.539
Smoking	54 (26.5)	59 (33.3)	1.897	0.170
Baseline NIHSS score, (x̄ ± s)	19.35 ± 10.27	21.18 ± 10.02	1.746	0.082
Cause of stroke (*n*, %)			2.891	0.236
Atherosclerotic	105 (52.2)	77 (43.5)		
Cardioembolic	82 (40.8)	86 (48.6)		
Other	14 (7.0)	14 (7.9)		
LDL (mmol/L, x̄ ± s)	2.470 ± 0.803	2.384 ± 0.748	−1.016	0.310
Glu (mmol/L, M, IQR)	7.697 ± 2.649	8.268 ± 3.081	1.886	0.060
HCY (μmol/L)	11.85 ± 8.356	12.24 ± 5.148	0.485	0.628
Cr (μmol/L)	74.63 ± 29.22	77.33 ± 41.89	0.732	0.456
mTICI 2b or 3 (*n*, %)	191 (95.0)	147 (83.7)	14.26	<0.001
ASITN/SIR<2 (*n*, %)	62 (31.0)	104 (58.8)	28.76	<0.001
Angioplasty and stenting (*n*, %)	38 (18.9)	30 (16.9)	0.244	0.621
Intravenous thrombolysis (*n*, %)	46 (22.8)	34 (19.2)	0.709	0.400
sICH (*n*, %)	12 (6.0)	39 (22.0)	20.80	<0.001
SAP (*n*, %)	64 (33.0)	102 (59.0)	24.89	<0.001

a *t* value for the independent-samples *t*-test comparing age between the two outcome groups.

OTR, onset-to-recanalization time; OTP, onset-to-puncture time; NIHSS, National Institutes of Health Stroke Scale; LDL, Low density lipoprotein; Glu, glucose; HCY, Homocysteine; Cr, Creatinine; mTICI, modified Thrombolysis in Cerebral Infarction; ASITN, American Society of Interventional and Therapeutic Neuroradiology; sICH, symptomatic Intracerebral Hemorrhage; SAP, Stroke-Associated Pneumonia.

As shown in Table 1, no statistically significant differences were found between the favorable and unfavorable outcome groups regarding age, sex, vascular risk factors, stroke subtype, intravenous thrombolysis, stent implantation, or other serum biomarkers (including low-density lipoprotein cholesterol, blood glucose, homocysteine, and creatinine) (all *p* > 0.05).

Successful recanalization was significantly more common in the favorable outcome group (95% vs. 83.7%, *p* < 0.001), while poor collateral circulation was observed more frequently in the unfavorable outcome group (58.5% vs. 31%, *p* < 0.001). Compared to the favorable outcome group, the unfavorable outcome group exhibited significantly higher incidence rates of symptomatic intracranial hemorrhage (22% vs. 6%, *p* < 0.001) and SAP (59% vs. 33%, *p* < 0.001).

### 3.2 Univariate and multivariate analyses of serum inflammatory factors for functional outcome in acute ischemic stroke

As shown in Table 2, compared to the favorable outcome group, the unfavorable outcome group exhibited significantly higher levels of white blood cell count (8.060 [6.480–10.735] vs. 7.975 [6.330–9.655], *p* < 0.05), neutrophil count (6.100 [4.480–8.925] vs. 5.925 [4.405–7.840], *p* < 0.05), monocyte count (0.506 ± 0.280 vs. 0.452 ± 0.211, *p* < 0.05), and NLR (4.970 [3.360–8.645] vs. 4.865 [2.677–7.672], *p* < 0.05). In contrast, the unfavorable outcome group had significantly lower LMR (2.989 ± 1.169 vs. 3.512 ± 1.843, *p* = 0.005) and PDW (16.00 [13.25–16.70] vs. 16.30 [15.57–16.60], *p* < 0.05).

**Table 2:** Association between inflammatory markers and clinical outcomes.

Inflammatory indicators	Favorable outcome (*n* = 201)	Unfavorable outcome (*n* = 177)	*t*, *Z or X^2^*	*p* value
WBC (10^9^/L, M, IQR)	7.975 (6.330–9.655)	8.060 (6.480–10.735)	2.184	0.030
Neu (10^9^/L, M, IQR)	5.925 (4.405–7.840)	6.100 (4.480–8.925)	2.270	0.024
Mon (10^9^/L, x̄ ± s)	0.452 ± 0.211	0.506 ± 0.280	2.072	0.039
Lym (10^9^/L, M, IQR)	3.183 (2.098–4.422)	2.766 (1.805–3.770)	−1.291	0.197
LMR (x̄ ± s)	3.512 ± 1.843	2.989 ± 1.169	−2.836	0.005
NLR (M, IQR)	4.865 (2.677–7.672)	4.970 (3.360–8.645)	2.382	0.018
PDW (%, M, IQR)	16.30 (15.57–16.60)	16.00 (13.25–16.70)	−2.027	0.044
SII (M, IQR)	757.56 (416.72–1378.36)	833.66 (468.93–1485.90)	1.378	0.169

WBC, White Blood Cell count; Neu, Neutrophil; Lym, Lymphocyte; Mon, Monocyte; LMR, Lymphocyte-to-Monocyte Ratio; NLR, Neutrophil-to-Lymphocyte Ratio; PDW, Platelet Distribution Width; SII, Systemic Immune-Inflammation Index.

Multivariate logistic regression was employed to assess the impact of factors that showed statistically significant differences in the univariate analysis on the functional outcome of AIS patients. As presented in Table 3, both LMR (OR 1.166, 95% CI 1.062–1.325, *p* < 0.05) and PDW (OR 1.093, 95% CI 1.001–1.193, *p* < 0.05) demonstrated statistically significant associations with functional outcome in AIS patients with AC-LVO.

**Table 3:** Logistic regression analysis of factors associated with functional outcome.

Variables	𝛽	SE	OR (95% CI )	*p* value
LMR	0.154	0.065	1.166 (1.026–1.325)	0.019
PDW	0.089	0.045	1.093 (1.001–1.193)	0.045

SE, Standard Error; OR, odds ratio; CI, Confidence Interval; LMR, Lymphocyte-to-Monocyte Ratio; PDW, Platelet Distribution Width; SII, Systemic Immune-Inflammation Index.

**Figure 2:**
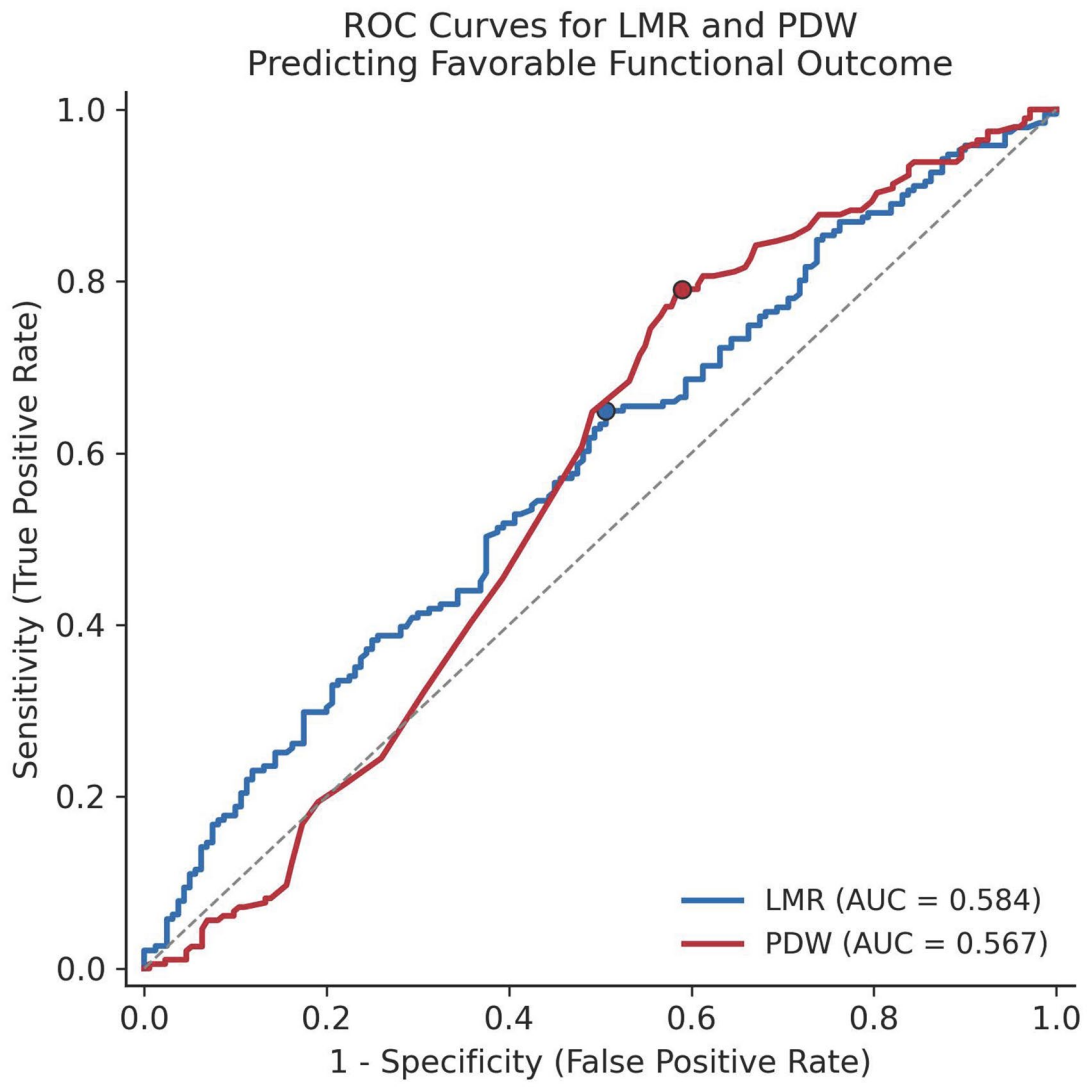
ROC curves for LMR and PDW in predicting favorable functional outcome. Dots indicate the optimal cut-off point (maximum Youden index) for each marker. AUC, area under the curve; LMR, Lymphocyte-to-Monocyte Ratio; PDW, Platelet Distribution Width.

To further characterize the discriminative performance of LMR and PDW, ROC curve analysis was performed (Table 4). The AUC for LMR was 0.578 (95% CI 0.517–0.639), with an optimal cut-off value of 2.69 (sensitivity 64.7%, specificity 48.8%). The AUC for PDW was 0.572 (95% CI 0.511–0.636), with an optimal cut-off value of 15.2% (sensitivity 78.9%, specificity 43.1%) (Figure 2). These AUC values indicate only modest discriminative ability, consistent with the relatively small absolute between-group differences observed in Table 2; LMR and PDW should therefore be regarded as adjunctive rather than stand-alone predictive markers.

**Table 4:** ROC curve analysis of LMR and PDW for predicting favorable functional outcome.

Inflammatory indicators	AUC (95% CI)	Optimal cut-off value	Sensitivity (%)	Specificity (%)
LMR	0.578 (0.517–0.639)	2.69	64.7	48.8
PDW (%)	0.572 (0.511–0.636)	15.2	78.9	43.1

Optimal cut-off values were determined by the maximum Youden index. AUC, area under the receiver operating characteristic curve; CI, confidence interval; LMR, Lymphocyte-to-Monocyte Ratio; PDW, Platelet Distribution Width.

**Figure 3:**
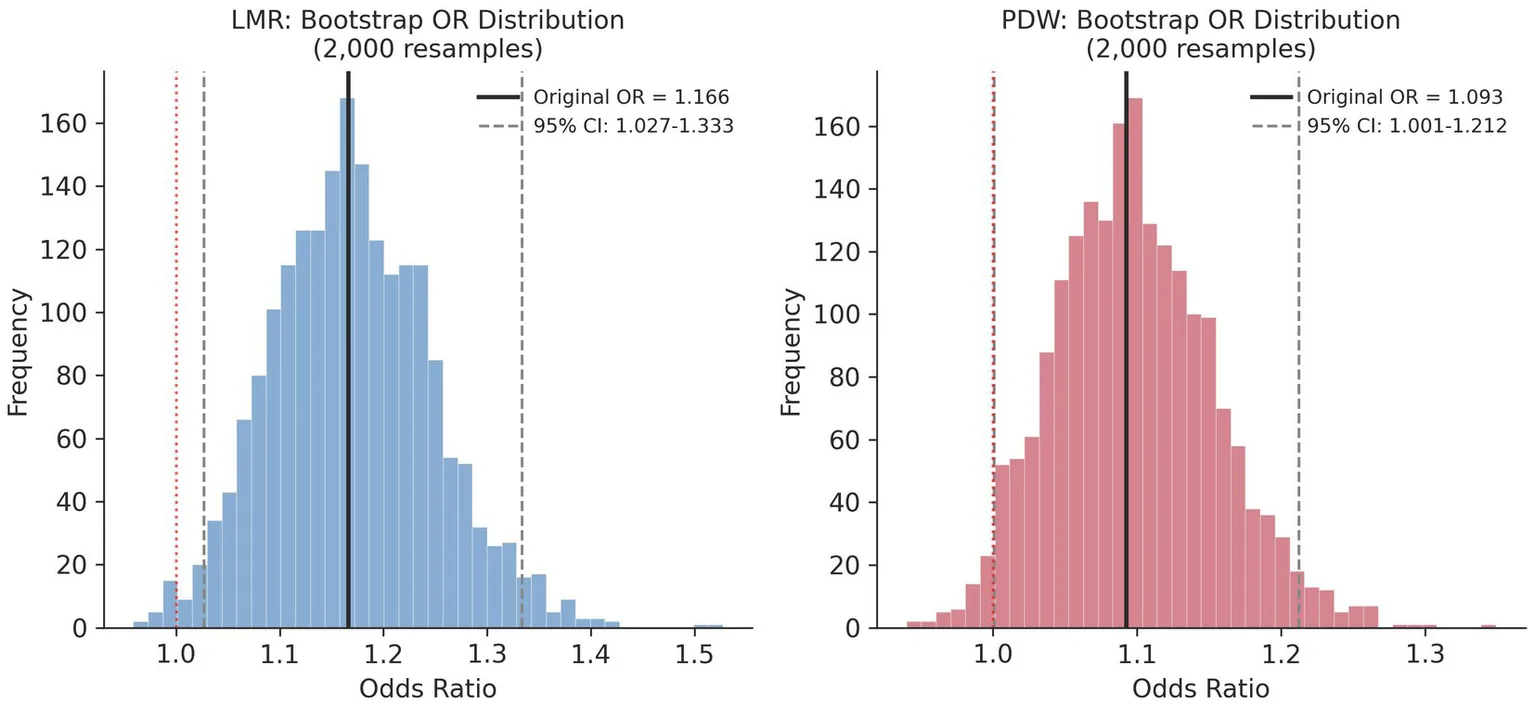
Bootstrap internal validation of the multivariable logistic regression model. Distribution of OR estimates for LMR and PDW across 2,000 bootstrap resamples. Solid black line, original model OR; dashed gray lines, bootstrap 95% CI; dotted red line, OR = 1 (null value).

Internal validation using bootstrap resampling (2,000 iterations) showed that the odds ratio estimates for LMR (median OR 1.168, 95% CI 1.027–1.333) and PDW (median OR 1.095, 95% CI 1.001–1.212) were stable and closely matched the original model estimates, supporting the internal validity of the multivariable model (Figure 3).

Because successful recanalization and favorable collateral circulation were markedly more common in the favorable-outcome group (Table 1) and are well-established strong predictors of stroke outcome, we performed a sensitivity analysis adding mTICI recanalization status, ASITN/SIR collateral grade, age, and baseline NIHSS score to the multivariable model. In this adjusted model, the point estimates for LMR (adjusted OR 1.130, 95% CI 0.982–1.299, *p* = 0.087) and PDW (adjusted OR 1.066, 95% CI 0.968–1.174, *p* = 0.191) were attenuated toward the null, while successful recanalization (OR 3.81, 95% CI 1.60–9.09) and favorable collateral status (OR 3.29, 95% CI 2.05–5.28) remained strong factors associated with outcome. This finding suggests that recanalization and collateral status may account for part of the univariate association between LMR/PDW and functional outcome; this residual confounding is discussed further as a study limitation. We additionally screened demographic and comorbidity variables (sex, atrial fibrillation, hypertension, hyperlipidaemia, diabetes, prior stroke, smoking, alcohol use, coronary artery disease) for association with functional outcome; none reached the pre-specified threshold of *p* < 0.10 for inclusion in the multivariable model (closest: atrial fibrillation, *p* = 0.15), consistent with the univariate results in Table 1.

As an additional robustness check for the confounding raised above, we performed a stratified analysis using the same confounders (mTICI recanalization status and ASITN/SIR collateral grade). LMR remained significantly higher in patients with favorable outcome within the successful-recanalization stratum (3.52 ± 1.83 vs. 3.07 ± 1.51, *p* = 0.020) and within the good-collateral stratum (3.57 ± 1.82 vs. 2.94 ± 1.58, *p* = 0.017), but not within the smaller failed-recanalization or poor-collateral strata, where statistical power was limited. PDW showed a similar borderline pattern (*p* = 0.07–0.08) in the poor-collateral and successful-recanalization strata but not in the others. These stratified findings suggest that the LMR-outcome association is not solely an artifact of confounding by recanalization or collateral status, although the small-sample strata remain inconclusive.

### 3.3 Univariate analysis of serum inflammatory factors for failed recanalization and symptomatic intracranial hemorrhage

The 378 patients were stratified according to recanalization status, and routine blood parameters were compared between the two groups. As shown in Table 5, compared to the successful recanalization group (mTICI 2b-3), the failed recanalization group (mTICI 0-2a) exhibited a significantly higher monocyte count (0.540 [0.335–0.710] vs. 0.425 [0.320–0.532], *p* < 0.05), while showing significantly lower levels of LMR (2.725 ± 1.894 vs. 3.339 ± 1.737, *p* < 0.05) and PDW (14.40 [13.05–16.60] vs. 16.300 [14.55–16.70], *p* < 0.05).

**Table 5:** Comparison of laboratory parameters between successful and failed recanalization groups.

Inflammatory indicators	Successful recanalization (*n* = 338)	Failed recanalization (*n* = 40)	*t*, *Z or X^2^*	*p* value
WBC (10^9^/L, x̄ ± s)	8.477 ± 3.104	9.299 ± 3.729	1.548	0.122
Neu (10^9^/L, M, IQR)	7.040 (4.740–10.63)	10.63 (6.405–11.69)	1.761	0.085
Mon (10^9^/L, M, IQR)	0.425 (0.320–0.532)	0.540 (0.335–0.710)	2.057	0.046
Lym (10^9^/L, x̄ ± s)	1.376 ± 0.744	1.275 ± 0.710	−0.820	0.431
LMR (x̄ ± s)	3.339 ± 1.737	2.725 ± 1.894	−2.014	0.045
NLR (x̄ ± s)	6.423 ± 5.432	7.566 ± 5.667	1.241	0.215
PDW (%, M, IQR)	16.300 (14.55–16.70)	14.40 (13.05–16.60)	−2.248	0.029
SII (M, IQR)	771.99 (428.02–1358.57)	1100.56 (688.14–1791.21)	1.597	0.117

WBC, White Blood Cell count; Neu, Neutrophil; Lym, Lymphocyte; Mon, Monocyte; LMR, Lymphocyte-to-Monocyte Ratio; NLR, Neutrophil-to-Lymphocyte Ratio; PDW, Platelet Distribution Width; SII, Systemic Immune-Inflammation Index.

Patients were stratified based on the occurrence of sICH, and serum index data were subsequently compared between the two groups. Relative to the non-sICH cohort, patients in the sICH group exhibited significantly lower serum LMR levels (2.784 [2.056–3.450] vs. 2.960 [1.965–4.199], *p* < 0.05; Table 6).

**Table 6:** Comparison of laboratory parameters between sICH and non-sICH groups.

Inflammatory indicators	sICH (*n* = 51)	Non-sICH (*n* = 327)	*t*, *Z or X^2^*	*p* value
WBC (10^9^/L, x̄ ± s)	8.603 ± 3.038	8.558 ± 3.207	−0.095	0.925
Neu (10^9^/L, x̄ ± s)	6.729 ± 2.852	6.664 ± 3.235	−0.135	0.892
Mon (10^9^/L, x̄ ± s)	0.469 ± 0.184	0.479 ± 0.255	0.271	0.787
Lym (10^9^/L, x̄ ± s)	1.268 ± 0.614	1.381 ± 0.758	0.997	0.319
LMR (M, IQR)	2.784 (2.056–3.450)	2.960 (1.965–4.199)	2.120	0.040
NLR (x̄ ± s)	7.116 ± 6.824	6.453 ± 5.255	−0.806	0.421
PDW (%, x̄ ± s)	15.31 ± 2.481	15.68 ± 2.439	1.022	0.307
SII (M, IQR)	796.29 (542.80–1148.37)	792.90 (420.43–1448.39)	0.057	0.957

WBC, White Blood Cell count; Neu, Neutrophil; Lym, Lymphocyte; Mon, Monocyte; LMR, Lymphocyte-to-Monocyte Ratio; NLR, Neutrophil-to-Lymphocyte Ratio; PDW, Platelet Distribution Width; SII, Systemic Immune-Inflammation Index; sICH, symptomatic Intracerebral Hemorrhage.

## 4 Discussion

The complete blood count (CBC), due to its cost-effectiveness and rapid availability, represents one of the most readily accessible laboratory tests in clinical practice. Composite inflammatory indices derived from the CBC, such as the NLR and LMR, have been demonstrated to reflect systemic inflammatory status and immunomodulatory balance more consistently than individual cell counts (6, 11). These indices have already shown prognostic association for prognosis across various cardiovascular and cerebrovascular diseases. Furthermore, the mechanistic link between PDW and unfavorable outcome is likely mediated through platelet activation-induced microvascular dysfunction and secondary inflammatory injury (12, 13).

Through systematic analysis of clinical data from patients with AC-LVO undergoing EVT, we found that systemic inflammatory factors and platelet activation status play a pivotal role in determining the ultimate neurological functional outcome of these patients. Unfavorable outcome was closely associated with heightened systemic inflammation and immune dysregulation. NLR and LMR are composite ratios derived from distinct combinations of inflammatory parameters. The joint analysis of these indices may provide additional insights into immune activity during the pathogenesis of ischemic stroke. Furthermore, since both integrated inflammatory ratios can be calculated directly from complete blood count results, they are relatively accessible biomarkers. Patients in the unfavorable outcome group exhibited significantly higher white blood cell counts, neutrophil counts, monocyte counts, and NLR values. This finding aligns with previously published research (14). Neutrophils are the first inflammatory cells to infiltrate the ischemic region during reperfusion injury. They directly exacerbate cerebral damage through the formation of neutrophil extracellular traps (NETs), microvascular occlusion, and the release of harmful substances (15). NLR reflects the innate immune response driven by neutrophils and the adaptive immune response supported by lymphocytes (16). Elevated NLR often manifests as an increase in neutrophils and a decrease in lymphocytes, with lymphocyte reduction indicating adaptive immune suppression, which may be directly related to an increased risk of post-stroke infections such as SAP (17).

A decrease in LMR results from a reduction in lymphocytes and/or an increase in monocytes, which indicates that the immune state is shifting toward a pro-inflammatory direction. The LMR combines lymphocyte and monocyte counts, reflecting two dimensions of the immune response: lymphocyte level represents adaptive immune capacity, while monocyte count reflects innate inflammatory burden. After acute stroke, the reduction and redistribution of lymphocytes, along with T cell apoptosis, weakens the anti-inflammatory defense mediated by regulatory T cells (Tregs) and increases susceptibility to post-stroke infections. At the same time, peripheral blood monocytes are recruited to the ischemic area through chemotactic signals such as CC-motif chemokine ligand 2 (CCL2), where they can exacerbate tissue damage by releasing matrix metalloproteinases (MMPs) and pro-inflammatory cytokines such as TNF-α and IL-1β, promoting blood–brain barrier disruption and secondary hemorrhagic transformation (5). In a retrospective study involving 121 patients who underwent mechanical thrombectomy (MT) for AIS, a lower LMR value at 24 h post-MT was significantly associated with unfavorable outcomes (18). A reduced LMR results from lymphocytopenia and/or monocytosis. Peripheral blood monocytes are significantly activated following spontaneous intracerebral hemorrhage (ICH). In a study by Wang et al., a lower LMR value was found to be associated with more severe ICH conditions on admission and an increased risk of case fatality during follow-up (19). Our results are partially consistent with previous studies. Subgroup analysis revealed that lower LMR values were present in patients with unfavorable outcomes, sICH, and failed recanalization (mTICI 0–2a). A low LMR may signify attenuated anti-inflammatory/immunoregulatory capacity (20), rendering the organism more susceptible to inflammatory cascades and consequently leading to more severe blood–brain barrier disruption and sICH.

Platelets play a key role in atherosclerosis and thrombogenesis. PDW reflects the heterogeneity of platelet volume in peripheral blood. Under conditions of acute stress, malignancy, or severe chronic inflammation, persistent low-grade platelet activation may lead to the consumption of specific platelet subpopulations or affect the differentiation and maturation of bone marrow megakaryocytes, ultimately resulting in decreased PDW (21–23). In this study, failed recanalization—associated with poor collateral circulation (ASITN/SIR < 2)—was characterized by lower LMR and PDW values. This suggests that collateral circulation status may not only influence blood supply to the ischemic core but also modulate the recruitment and clearance of inflammatory cells, with both processes jointly determining the extent of cerebral tissue damage. We observed lower PDW in patients with unfavorable outcomes and in those with failed recanalization, which may be linked to microvascular dysfunction and persistent platelet consumption. However, a study on carotid artery stenosis reported that a higher PDW was significantly associated with greater stenosis severity (24). The precise mechanisms underlying these observations warrant further investigation.

Although LMR and PDW were statistically significant factors associated with outcome in this study, their odds ratios were close to 1, indicating limited clinical impact as prognostic markers. Accordingly, we suggest using them as supplementary risk stratification tools rather than as strong factors independently associated with outcome. Their added value beyond conventional clinical factors—including age, baseline stroke severity, and collateral circulation—needs further prospective validation.

Several additional limitations warrant explicit acknowledgment. First, ROC analysis indicated only modest discriminative ability for both LMR (AUC 0.578) and PDW (AUC 0.572), reinforcing that these indices are best regarded as adjunctive, rather than stand-alone, prognostic tools. Second, because successful recanalization and favorable collateral circulation were considerably more frequent in the favorable-outcome group, a sensitivity analysis additionally adjusting for these factors, together with age and baseline NIHSS score, attenuated the associations of LMR and PDW with outcome toward the null; this indicates that residual confounding by recanalization and collateral status cannot be fully excluded. A complementary stratified analysis by recanalization and collateral status showed that the LMR-outcome association held within the larger successful-recanalization and good-collateral strata but was not confirmed within the smaller strata, indicating that confounding by these factors is unlikely to fully explain the LMR association, though this cannot be considered definitive given the limited power of the small strata. Third, our dataset did not capture whether patients received any specific systemic anti-inflammatory or immunomodulatory treatment during hospitalization, so we cannot determine whether such treatment influenced the observed associations. Fourth, LMR and PDW were measured only once at baseline; dynamic, serial monitoring may better capture the evolving inflammatory trajectory and its relationship with outcome. Finally, although bootstrap resampling supported the internal stability of our estimates, external validation in independent, multicenter, prospective cohorts is still required before LMR and PDW can be recommended for clinical risk stratification.

## 5 Conclusion

The findings of this study indicate that in patients with AC-LVO stroke undergoing EVT, pre-existing systemic hyperinflammation and immune dysregulation are significant factors associated with unfavorable functional outcome. Elevated NLR, reduced LMR, and decreased PDW may provide supplementary information for risk stratification for unfavorable functional recovery following EVT. Specifically, a low LMR is significantly associated with an increased risk of symptomatic intracranial hemorrhage. Furthermore, a high monocyte count combined with low LMR and PDW levels is closely linked to failed recanalization.

These readily accessible, CBC-derived indices effectively reflect a patient’s preprocedural inflammatory and immune status. They may have potential utility when incorporated into multimodal risk assessment models together with established clinical value and imaging factors. Future research should focus on developing multimodal predictive models and investigating the feasibility of dynamically monitoring these biomarkers to guide personalized immunomodulatory therapy.

## Data Availability

The original contributions presented in the study are included in the article/supplementary material, further inquiries can be directed to the corresponding authors.
